# Astrocytes Mediate In Vivo Cholinergic-Induced Synaptic Plasticity

**DOI:** 10.1371/journal.pbio.1001259

**Published:** 2012-02-14

**Authors:** Marta Navarrete, Gertrudis Perea, David Fernandez de Sevilla, Marta Gómez-Gonzalo, Angel Núñez, Eduardo D. Martín, Alfonso Araque

**Affiliations:** 1 Instituto Cajal, Consejo Superior de Investigaciones Científicas. Madrid, Spain; 2 Department Anatomía, Histología y Neurociencia, Facultad de Medicina, Universidad Autónoma de Madrid, Madrid, Spain; 3 Laboratory of Neurophysiology and Synaptic Plasticity, Albacete Science and Technology Park (PCyTA), Institute for Research in Neurological Disabilities (IDINE), University of Castilla-La Mancha, Albacete, Spain; University of Basel, Switzerland

## Abstract

Long-term potentiation (LTP) of synaptic transmission represents the cellular basis of learning and memory. Astrocytes have been shown to regulate synaptic transmission and plasticity. However, their involvement in specific physiological processes that induce LTP in vivo remains unknown. Here we show that in vivo cholinergic activity evoked by sensory stimulation or electrical stimulation of the septal nucleus increases Ca^2+^ in hippocampal astrocytes and induces LTP of CA3-CA1 synapses, which requires cholinergic muscarinic (mAChR) and metabotropic glutamate receptor (mGluR) activation. Stimulation of cholinergic pathways in hippocampal slices evokes astrocyte Ca^2+^ elevations, postsynaptic depolarizations of CA1 pyramidal neurons, and LTP of transmitter release at single CA3-CA1 synapses. Like in vivo, these effects are mediated by mAChRs, and this cholinergic-induced LTP (c-LTP) also involves mGluR activation. Astrocyte Ca^2+^ elevations and LTP are absent in IP_3_R2 knock-out mice. Downregulating astrocyte Ca^2+^ signal by loading astrocytes with BAPTA or GDPβS also prevents LTP, which is restored by simultaneous astrocyte Ca^2+^ uncaging and postsynaptic depolarization. Therefore, cholinergic-induced LTP requires astrocyte Ca^2+^ elevations, which stimulate astrocyte glutamate release that activates mGluRs. The cholinergic-induced LTP results from the temporal coincidence of the postsynaptic activity and the astrocyte Ca^2+^ signal simultaneously evoked by cholinergic activity. Therefore, the astrocyte Ca^2+^ signal is necessary for cholinergic-induced synaptic plasticity, indicating that astrocytes are directly involved in brain storage information.

## Abstract 2

In vivo and in vitro studies reveal that astrocytes, classically considered supportive cells for neurons, regulate synaptic plasticity in the mouse hippocampus and are directly involved in information storage.

## Author Summary

Information processing in the brain was classically thought to rely solely on neurons, whereas astrocytes, the most abundant glial cells in the brain, were considered supportive cells for neurons. However, astrocytes are known to respond to neuronal signals and regulate the function of synapses, so they may indeed serve active roles during information processing and storage in the brain. We investigated whether these phenomena occur in vivo and whether astrocytes participate in synaptic plasticity and long-term potentiation (LTP), which are thought to represent the cellular basis of learning and memory. We found that sensory stimulation in rodents triggers the activity of neurons that release the neurotransmitter acetylcholine and activates astrocytes in the hippocampus, a brain region involved in learning and memory. Acetylcholine elevates intracellular calcium in astrocytes, which then stimulates the release of glutamate, another neurotransmitter. Glutamate released from astrocytes acts on synaptic receptors, increasing synaptic strength and leading to LTP of the efficacy of transmission in synapses. This is the first demonstration of the direct involvement of astrocytes in the generation of in vivo LTP. We suggest that astrocytes are cellular sources of signals underlying synaptic plasticity and are directly involved in memory processes and brain information storage.

## Introduction

Compelling evidence obtained by different groups during the last years indicate that astrocytes play important roles in synaptic function [1]–[4]. In addition to their well-known passive homeostatic control of synaptic function, astrocytes sense synaptic activity responding with Ca^2+^ elevations to synaptically released neurotransmitters and, in turn, release gliotransmitters that regulate synaptic transmission and plasticity [5]–[14]. This evidence has led to the establishment of the Tripartite Synapse concept, in which astrocytes actively exchange information with the neuronal synaptic elements, suggesting that astrocytes may be considered as integral elements of the synapses being directly involved in synaptic physiology [1]–[4]. While this evidence has been largely obtained in brain slices, recent in vivo studies that used transgenic mice in which the gliotransmitter release of ATP was impaired have shown the participation of astrocytes in certain cortical network activity and in animal behaviour [2],[3],[15],[16]. However, the exact underlying cellular mechanisms are largely undefined. Furthermore, while the involvement of astrocytes in some forms of long-term potentiation (LTP) has been shown in hippocampal slices (e.g., [6],[11]), the active participation of astrocytes in specific forms of synaptic plasticity in vivo remains unknown.

Cholinergic system is involved in many different processes of brain function [17]. In the hippocampus, cholinergic activity modulates neuronal excitability [18], network activity [19], as well as synaptic transmission and plasticity [20],[21]. In the CA1 region, acetylcholine (ACh) induces CA1 pyramidal neuron depolarization [18], theta rhythm generation [19], and LTP of glutamatergic CA3-CA1 synaptic transmission [20],[21], as well as astrocyte Ca^2+^ elevations [22],[23]. However, the physiological meaning of the cholinergic evoked astrocyte Ca^2+^ signal remains unknown.

In the present work we have investigated two fundamental questions regarding the direct involvement of astrocytes in synaptic physiology, i.e, whether astrocytes actively participate in physiological processes underlying synaptic plasticity, and whether astrocyte synaptic modulation occurs in vivo. We have recently shown that the coincidence of astrocyte Ca^2+^ elevations evoked by Ca^2+^ uncaging and mild postsynaptic depolarization induces LTP in hippocampal synapses [11]. Therefore, we have investigated whether the astrocyte Ca^2+^ signal evoked by cholinergic activity [22] is involved in the generation of cholinergic-induced LTP of glutamatergic CA3-CA1 synapses.

Using in vivo experimental approaches, we have found that cholinergic activity evoked by sensory stimulation or electrical stimulation of the septal nucleus, the main cholinergic input to the hippocampus, elevated Ca^2+^ in hippocampal astrocytes and induced LTP in CA3-CA1 synapses. Using hippocampal slices to investigate the underlying cellular mechanisms, we have found that stimulation of cholinergic axons evoked astrocyte Ca^2+^ elevations, depolarization of CA1 pyramidal neurons, and LTP in CA3-CA1 synapses. Like in vivo, astrocyte Ca^2+^ elevations and LTP required mAChR activation, and LTP also required mGluR activation. Cholinergic-induced astrocyte Ca^2+^ elevations and LTP were absent both in IP_3_R2 knock-out mice and in wildtype mice after loading astrocytes with BAPTA or GDPβS (which prevented astrocyte Ca^2+^ signalling). Notably, LTP was rescued by simultaneous astrocyte Ca^2+^ uncaging and postsynaptic depolarization. Taken together, these results indicate that astrocyte Ca^2+^ signal is necessary for cholinergic-induced hippocampal synaptic plasticity.

In summary, present results show that cholinergic LTP requires the astrocyte Ca^2+^ signal, which stimulates the release of glutamate from astrocytes that activates mGluRs on neurons. Then, cholinergic-induced hippocampal LTP results from the coincidence of astrocyte and postsynaptic activities simultaneously evoked by cholinergic signalling.

## Results

### Cholinergic Activity Evokes Astrocyte Ca^2+^ Elevations and LTP In Vivo

**Figure 1:**
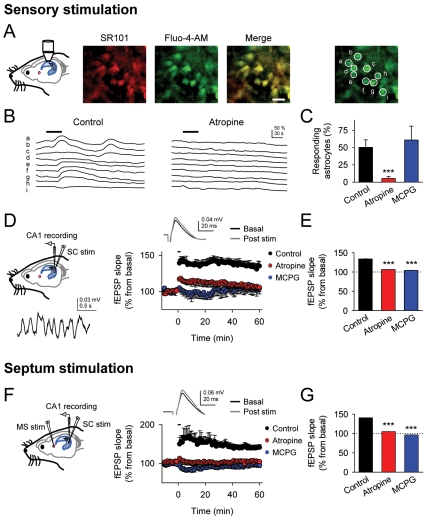
Cholinergic activity induces astrocyte Ca^2+^ elevations and LTP in CA3-CA1 synapses in the hippocampus in vivo. (A) Schematic drawing of the experimental approach used to monitor Ca^2+^ levels in hippocampal astrocytes in vivo; representative images of astrocytes labeled with sulforhodamine 101 (SR101) and loaded with Fluo-4-AM; corresponding merge image; and image of Fluo-4-loaded astrocytes displaying regions of interests. Scale bar, 20 µm. (B) Fluorescence traces of Ca^2+^ levels in regions of interests in astrocytes showed in (A) evoked by tail pinch sensory stimulation (horizontal bars) in control and in the presence of atropine. (C) Proportion of astrocytes responding to sensory stimulation in control (66 astrocytes from *n* = 8 rats), atropine (32 astrocytes from *n* = 4 rats), and MCPG (15 astrocytes from *n* = 3 rats). (D) Schematic drawing of the in vivo experimental approach showing the stimulating electrode in the Schaffer collaterals (SC) and the extracellular recording electrode of fEPSPs placed in the hippocampal CA1 region, and a representative trace of a field potential showing hippocampal theta rhythm activity (bottom) during tail pinch sensory stimulation. Right, Relative fEPSP slope (from basal values) versus time. Zero time corresponds to the onset of stimulation (as in all other figures). Inset: mean fEPSPs before and 60 min after stimulation. (E) Average relative changes of fEPSP evoked 60 min after sensory stimulation in control (*n* = 7), atropine (*n* = 6), and MCPG (*n* = 6). (F) Schematic drawing showing the additional stimulating electrode in the medial septum nucleus. Right, Relative fEPSP slope (from basal values) versus time. Zero time corresponds to the onset of stimulation that lasted 90.7 s (horizontal bar). Inset: mean fEPSPs before and 60 min after septum stimulation. (G) Average relative changes of fEPSP evoked 60 min after stimulation in control (*n* = 9), atropine (*n* = 6), and MCPG (*n* = 6). ***p<0.001. Data are presented as means ± s.e.m (as in all other figures).

We first assessed in vivo whether cholinergic activity regulates astrocyte Ca^2+^ signal and synaptic transmission (see Materials and Methods). In anesthetized rats, somatosensory stimulation by tail pinch, which stimulates cholinergic activity and hippocampal theta rhythm [24],[25], evoked Ca^2+^ elevations in hippocampal astrocytes (34 out of 66 astrocytes from *n* = 8 rats) that were abolished by the cholinergic muscarinic receptor (mAChRs) antagonist atropine (5 mg/kg) (*n* = 4 rats; Figure 1A–C). We analyzed hippocampal synaptic transmission in CA3-CA1 synapses, recording field EPSPs (fEPSPs) evoked by Schaffer collaterals (SC) stimulation in the CA1 pyramidal layer. Sensory stimulation also induced the LTP of fEPSPs (*n* = 7; Figure 1D and 1E). Similar LTP was also found after electrical stimulation of the medial septal nucleus (the main cholinergic input to the hippocampus) with a theta-like burst stimulation paradigm (TBS) (*n* = 9; see Materials and Methods; Figure 1F and 1G) [26],[27]. This LTP evoked by sensory or electrical stimulation was prevented in the presence of antagonists of either muscarinic receptors (mAChRs; 5 mg/Kg atropine) or metabotropic glutamate receptors (mGluRs; 1 mM MCPG) (*n* = 6 in each case) (Figure 1D–G), indicating that septohippocampal cholinergic activity induced the long-term potentiation (c-LTP) of CA3-CA1 synapses, which also required mGluR activation. Furthermore, because astrocyte responsiveness to sensory stimulation was similar in control and in the presence of MCPG (*n* = 3; Figure 1C), mGluR activation was downstream the astrocyte Ca^2+^ signal.

### Cholinergic-Induced LTP Requires mGluR Activation

**Figure 2:**
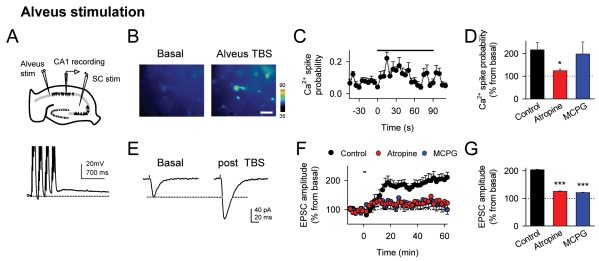
Cholinergic activity in hippocampal slices induces astrocyte Ca^2+^ elevations and LTP in CA3-CA1 synapses. (A) Schematic drawing showing the stimulating electrodes (alveus and SC) and the whole-cell recording electrode (CA1 pyramidal neuron) in hippocampal slices, and a representative postsynaptic response (bottom) to one train of alveus TBS (action potentials evoked by TBS were truncated). (B) Pseudocolor images representing fluorescence intensities of fluo-4-filled astrocytes before and during alveus stimulation. Scale bar, 40 µm. (C) Astrocyte Ca^2+^ spike probability versus time. (D) Average relative changes of maximum astrocyte Ca^2+^ spike probability (from basal values) during alveus stimulation in control (132 astrocytes from *n* = 13 slices), atropine (94 astrocytes from *n* = 10 slices), and MCPG (71 astrocytes from *n* = 7 slices). (E) Mean EPSCs before and 60 min after alveus stimulation. (F) Relative EPSC amplitudes (from basal values) versus time. (G) Average relative changes of EPSC amplitudes evoked 60 min after stimulation in control (*n* = 13), atropine (*n* = 10), and MCPG (*n* = 12). In (C) and (F), zero time corresponds to the onset of stimulation that lasted 90.7 s (horizontal bars). *p<0.05, ***p<0.001. Data are presented as means ± s.e.m.

We then investigated the cellular mechanisms underlying c-LTP. Using rat hippocampal slices, we simultaneously monitored EPSCs evoked by SC stimulation in CA1 pyramidal neurons and intracellular Ca^2+^ levels in *stratum radiatum* astrocytes. After basal control recordings, we stimulated afferent pathways in the alveus, which contains cholinergic axons from the medial septal nucleus [22],[28]. To prevent possible NMDAR-mediated synaptic plasticity, experiments were performed in the presence of the NMDAR antagonist AP5 (50 µM). Alveus stimulation with TBS evoked transient postsynaptic depolarizations of CA1 pyramidal neurons (12.6±1.7 mV; *n* = 13, Figure 2A), astrocyte Ca^2+^ elevations (*n* = 132 astrocytes from 13 slices), and c-LTP of CA3-CA1 synaptic transmission (*n* = 13; Figure 2A–F). The c-LTP was accompanied by a reduction of the paired-pulse facilitation index (0.43±0.05 in basal and 0.35±0.04 60 min after TBS; *n* = 10; *p* = 0.017; Figure S1), which is consistent with a presynaptic mechanism of action.

Both astrocyte Ca^2+^ elevations and c-LTP were reduced in the presence of 50 µM atropine (*n* = 94 astrocytes from 10 slices; *n* = 10 neurons; Figure 2D and 2G), indicating the involvement of cholinergic muscarinic receptors and confirming that c-LTP was also present in hippocampal slices. Furthermore, MCPG (1 mM) prevented c-LTP (*n* = 12; Figure 2G), without affecting TBS-induced postsynaptic depolarization (in control: 12.6±1.7 mV; *n* = 10; in MCPG: 10.4±2.1 mV, *n* = 7, *p* = 0.41) and astrocyte Ca^2+^ elevations (*n* = 71 astrocytes from 7 slices; Figure 2D) (cf., [22],[23]), indicating that c-LTP also requires mGluR activation.

### Astrocyte Ca^2+^ Elevations Are Required for Cholinergic-Induced LTP

**Figure 3:**
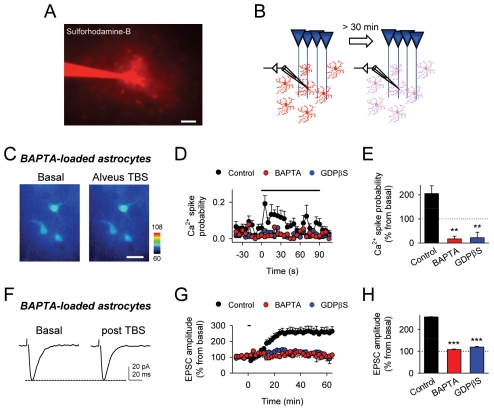
Cholinergic-induced hippocampal LTP requires astrocyte Ca^2+^ elevations. (A) Fluorescence image showing dialysis of sulforhodamine B into the astrocytic network after loading a single astrocyte with the dye (1 mg/ml) through the whole-cell recording pipette. Scale bar, 40 µm. (B) Schematic drawing depicting BAPTA or GDPβS dialysis into the astrocytic network from the recorded astrocyte. (C) Pseudocolor images representing fluorescence intensities of fluo-4- and BAPTA-filled astrocytes before (basal) and during alveus stimulation. Scale bar, 20 µm. (D) Astrocyte Ca^2+^ spike probability in control, BAPTA-, and GDPβS-loaded astrocytes. (E) Average relative changes of maximum astrocyte Ca^2+^ spike probability (from basal values) during alveus stimulation in control (100 astrocytes from *n* = 11 slices), BAPTA- (96 astrocytes from *n* = 10 slices), and GDPβS-loaded astrocytes (76 astrocytes from *n* = 10 slices). (F) Mean EPSCs (*n* = 10 consecutive EPSCs) before and 60 min after alveus TBS in a slice with BAPTA-loaded astrocytes. (G) Relative EPSC amplitudes versus time in control and BAPTA- and GDPβS-loaded astrocytes. (H) Average relative changes of EPSC amplitudes evoked 60 min after alveus TBS in control (*n* = 8), BAPTA- (*n* = 7), and GDPβS-loaded astrocytes (*n* = 5). In (D) and (G), zero time corresponds to the onset of stimulation that lasted 90.7 s (horizontal bars). **p<0.01, ***p<0.001. Data are presented as means ± s.e.m.

We have recently shown that the coincidence of Ca^2+^ uncaging-evoked glutamate release from astrocytes and a mild postsynaptic depolarization of CA1 pyramidal neurons induced LTP at CA3-CA1 synapses through presynaptic mGluR activation [11]. Therefore, we then investigated whether c-LTP required astrocyte Ca^2+^ elevations by analyzing the consequences of the dialysis of either BAPTA (which chelates intracellular Ca^2+^) or GDPβS (which prevents G protein-mediated intracellular signaling) into the astrocytic network (Figure 3A and 3B). We recorded single astrocytes including either BAPTA (40 mM) or GDPβS (20 mM) in the whole-cell recording pipette. These substances are known to spread to a large number of gap-junction connected hippocampal astrocytes [7],[14],[29]–[31]. In either BAPTA- and GDPβS-loaded astrocytes, astrocyte Ca^2+^ elevations evoked by alveus TBS were prevented in an area at least 150 µm around the recorded astrocyte (Figure 3C–E), without significantly affecting the postsynaptic depolarization (11.9±1.4 mV and 11.4±2.3 mV, *n* = 7 and 5, respectively; compared with 12.6±1.7 mV; *n* = 10, in control; *p* = 0.74 and *p* = 0.67). Simultaneous recordings of CA3-CA1 EPSCs showed that c-LTP was also prevented (Figure 3F–H), indicating that G-protein-mediated astrocyte Ca^2+^ signaling is necessary for c-LTP induction, and supporting the idea that c-LTP results from mGluR activation induced by Ca^2+^-dependent glutamate release from astrocytes.

**Figure 4:**
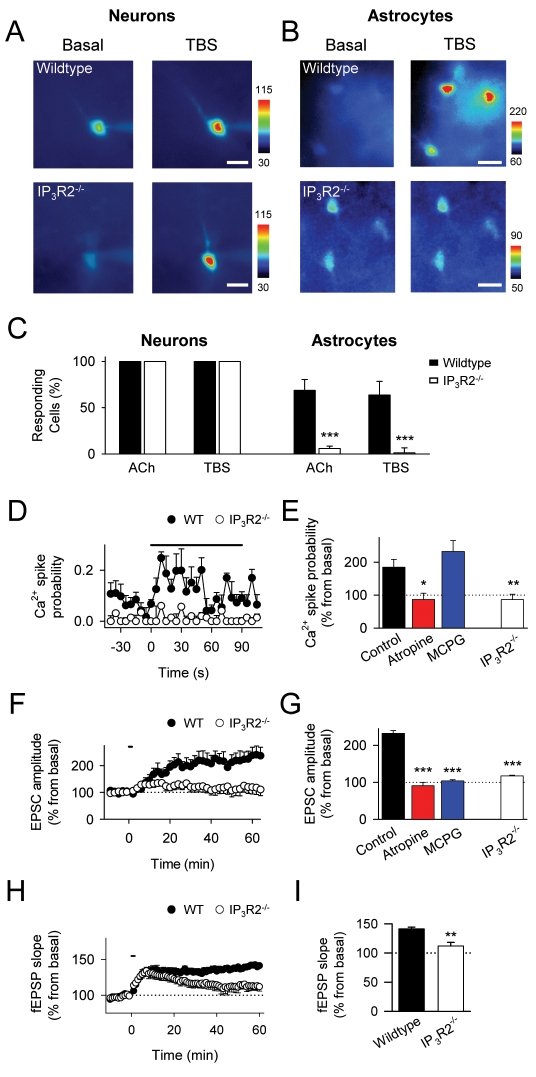
Cholinergic-induced hippocampal LTP is altered in IP3R2^−/−^ mice. (A) Pseudocolor images representing fluorescence intensities of pyramidal neurons filled with fuo-4 through the recording pipette before (basal) and during alveus TBS in wildtype (top) and IP_3_R2^−/−^ mice (bottom). Scale bar, 20 µm. (B) Pseudocolor images representing fluorescence intensities of fluo-4-filled astrocytes before (basal) and during alveus TBS in wildtype (top) and IP_3_R2^−/−^ mice (bottom). Scale bar, 15 µm. (C) Proportion of responding neurons and astrocytes to ACh application and alveus TBS in wildtype and IP_3_R2^−/−^ mice (6 and 10 neurons from *n* = 6 and 10 slices for each stimulus in wildtype and IP_3_R2^−/−^ mice, respectively; for ACh: 111 and 157 astrocytes from *n* = 6 and 15 slices; for TBS: 81 and 64 astrocytes from *n* = 9 and 10 slices, in wildtype and IP_3_R2^−/−^ mice, respectively). (D) Astrocyte Ca^2+^ spike probability in wildtype and IP_3_R2^−/−^ mice (81 and 64 astrocytes from *n* = 9 and 10 slices, respectively). (E) Average relative changes of maximum astrocyte Ca^2+^ spike probability (from basal values) during alveus stimulation in control (81 astrocytes from *n* = 9 slices), atropine (25 astrocytes from *n* = 4 slices), and MCPG (40 astrocytes from *n* = 5 slices) in wildtype mice and control IP_3_R2^−/−^ mice (64 astrocytes from *n* = 10 slices). (F) Relative EPSC amplitudes versus time in slices from wildtype (*n* = 8) and IP_3_R2^−/−^ (*n* = 8) mice. (G) Average relative changes of EPSC amplitudes evoked 60 min after alveus TBS in slices from wildtype mice in control (*n* = 8), atropine (*n* = 4), and MCPG (*n* = 5), and from IP_3_R2^−/−^ mice (*n* = 8). (H) Relative mean fEPSP slope versus time in *in vivo* wildtype (*n* = 6) and IP_3_R2^−/−^ mice (*n* = 4) before and after sensory stimulation. (I) Average relative changes of the mean fEPSP slope evoked 60 min after sensory stimulation in wildtype (*n* = 6) and IP_3_R2^−/−^ mice (*n* = 6). In (D), (F), and (H), zero time corresponds to the onset of stimulation (horizontal bars). *p<0.05, **p<0.01, ***p<0.001.

To further test this idea, we analyzed the phenomena in slices from wildtype and inositol-1,4,5-trisphosphate(IP_3_)-receptor type 2-deficient mice (IP_3_R2^−/−^) [32], which is the primary functional IP_3_R expressed by astrocytes that mediate intracellular Ca^2+^ mobilization [33]. In agreement with this report, local application of ACh increased intracellular Ca^2+^ in all CA1 pyramidal neurons tested in both wildtype and IP_3_R2^−/−^ mice (6 and 10 neurons from *n* = 6 and 10 slices, respectively; Figure 4C) and induced Ca^2+^ elevations in most astrocytes from wildtype animals but not from knockout mice (78 out of 111 and 13 out of 157 astrocytes from *n* = 6 and 15 slices, respectively; *p*<0.001; Figure 4C). Alveus TBS stimulation also elevated Ca^2+^ in neurons from both wildtype and IP_3_R2^−/−^ mice (6 and 10 neurons from *n* = 6 and 10 slices, respectively; Figure 4A and 4C). In wildtype mice, alveus TBS induced astrocyte Ca^2+^ elevations (62 out of 81 astrocytes from *n* = 9 slices), which were abolished by atropine (14 out of 25 astrocytes from *n* = 4 slices responded to TBS in control but not in atropine) (Figure 4C–E), and evoked c-LTP (*n* = 8; Figure 4F and 4G), which was prevented by atropine (*n* = 4) or MCPG (*n* = 5; Figure 4G). In contrast, both astrocyte Ca^2+^ elevations (64 astrocytes from *n* = 10 slices) and c-LTP induced by alveus TBS were largely prevented in IP_3_R2^−/−^ mice (*n* = 8; Figure 4B–G) confirming the requirement of astrocyte Ca^2+^ signaling for c-LTP.

Consistent with these results, the LTP observed in vivo after sensory stimulation in wildtype mice (*n* = 6) was strongly diminished in IP_3_R2^−/−^ mice (*n* = 6) (Figure 4H and 4I). The presence of the initial potentiation and the residual LTP observed in transgenic mice suggest that additional synaptic plasticity mechanisms may also be present in vivo, where the phenomenon could not be pharmacologically isolated as it was in vitro (see Discussion).

Taken together, these results indicate that astrocyte Ca^2+^ elevations play a significant role in the cholinergic-induced LTP.

### Postsynaptic Depolarizations Are Necessary for Cholinergic-Induced LTP

**Figure 5:**
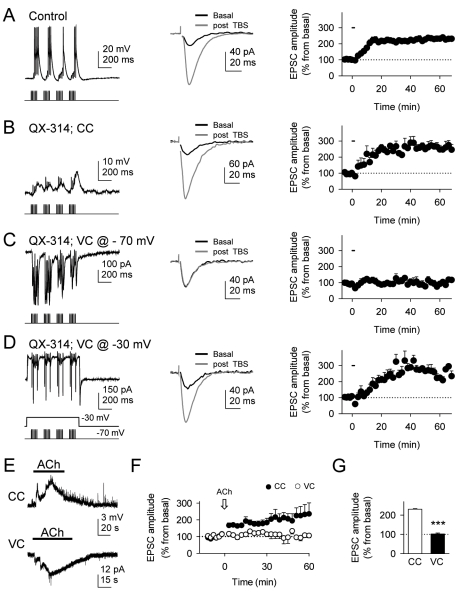
Cholinergic-induced hippocampal LTP depends on mild postsynaptic depolarizations. (A) Left, CA1 pyramidal neuron response to a train of alveus TBS recorded in current-clamp conditions. Center, mean EPSCs (*n* = 10 consecutive EPSCs) before and 60 min after alveus TBS. Right, relative EPSC amplitudes versus time (*n* = 6). Zero time corresponds to the onset of alveus TBS that lasted 90.7 s (horizontal bar). (B, C, and D) as in (A), but in QX-314-loaded neuron recorded in current-clamp conditions (*n* = 7), in voltage-clamp conditions at a holding potential of −70 mV (*n* = 5) and −30 mV (*n* = 5), respectively. (E) Representative neuronal responses to application of ACh in current- (CC) and voltage-clamp (VC) conditions at a holding potential of −70 mV. (F) Relative EPSC amplitudes versus time in CC and VC before and after ACh application (arrow). (G) Average relative changes of EPSC amplitudes evoked 60 min after ACh application in CC and VC (*n* = 10 and 10, respectively). ***p<0.001.

We next investigated whether the cholinergic-induced postsynaptic activity is required for c-LTP generation. We performed simultaneous paired-recordings from two pyramidal neurons that were loaded through the recording pipette with 5 mM QX314 that intracellularly blocks Na^+^-mediated action potentials. In QX314-loaded neurons, TBS-induced action potential firing was absent, but low-amplitude mild depolarizations (10.4±2.1 mV; *n* = 7) and c-LTP were still present (*n* = 7) (Figure 5A and 5B), indicating that c-LTP does not require postsynaptic action potentials. However, preventing the neuronal depolarization by holding the postsynaptic cell at −70 mV in voltage-clamp conditions abolished c-LTP (*n* = 5) (Figure 5C), while it was preserved in adjacent neurons recorded under current clamp conditions (*n* = 5), which displayed TBS-induced postsynaptic depolarizations. Furthermore, in voltage-clamped neurons, c-LTP was rescued by pairing alveus TBS with a postsynaptic mild depolarization to −30 mV (*n* = 5; Figure 5D). The TBS-induced astrocyte Ca^2+^ signal was unaffected by these postsynaptic manipulations (not shown).

To further confirm the involvement of cholinergic receptors in the postsynaptic depolarization required for c-LTP, we applied ACh to directly activate astrocytic and postsynaptic receptors while recording pyramidal neurons in either current- or voltage-clamp conditions. While ACh evoked postsynaptic depolarizations and induced LTP in current-clamped neurons, it failed to induce LTP in voltage-clamped neurons (Figure 5E–G). Although a partial contribution of glutamatergic signaling cannot be discarded, these results are consistent with the idea that cholinergic activity is responsible for the postsynaptic depolarization required for c-LTP.

Taken together, these results indicate that c-LTP requires both the postsynaptic depolarization and the astrocyte Ca^2+^ elevations.

### Cholinergic-Induced LTP Results from the Coincidence of Astrocyte Ca^2+^ Signal and Postsynaptic Activity

**Figure 6:**
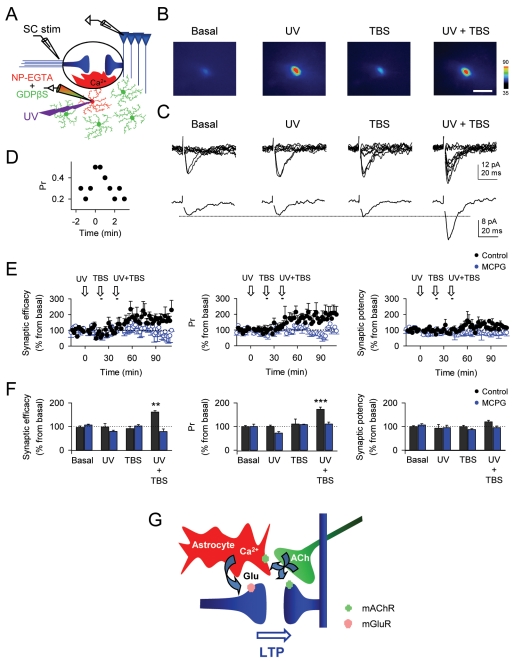
Astrocyte Ca^2+^ elevations induce LTP of transmitter release at single hippocampal synapses. (A) Schematic drawing depicting simultaneous recordings from one pyramidal neuron, one astrocyte filled with NP-EGTA and GDPβS filling the astrocytic network, and the stimulating electrode. (B) Pseudocolor images representing fluorescence intensity of a NP-EGTA- and fluo-4-filled astrocyte before (basal) and during UV-flash astrocyte stimulation, alveus TBS, and pairing both stimuli. Scale bar, 20 µm. (C) Synaptic responses (10 consecutive stimuli; top traces) and corresponding average EPSCs (bottom traces) obtained from paired whole-cell recordings before (basal) and 20 min after UV-flash astrocyte stimulation, alveus TBS, and pairing both stimuli. (D) Representative transient increase of the probability of neurotransmitter release (Pr) (bin width, 0.5 min) evoked by UV-flash astrocyte stimulation in a single synapse. Zero time corresponds to the time of UV stimulation. (E) Relative changes in synaptic efficacy (i.e., mean amplitude of responses including successes and failures of neurotransmission), probability of neurotransmitter release (Pr), and synaptic potency (i.e., mean EPSC amplitude excluding failures) (bin width, 2 min) over time evoked by UV-flash astrocyte stimulation, alveus TBS, and pairing both stimuli in control (*n* = 9) and MCPG (*n* = 6). (F) Relative changes of synaptic parameters evoked 20–30 min after UV-flash astrocyte stimulation, alveus TBS, and pairing both stimuli in control and MCPG (*n* = 9 and 6 astrocyte-neuron pairs, respectively). **p<0.01, ***p<0.001. (G) Schematic drawing representing the astrocyte-mediated cholinergic-induced LTP. Cholinergic axons simultaneously activate AChRs in pyramidal neurons and astrocytes. Consequent astrocyte Ca^2+^ elevations stimulate glutamate (Glu) release that increases transmitter release probability through presynaptic mGluR activation. The temporal coincidence of astrocyte and postsynaptic activities simultaneously evoked by cholinergic activity induces long-term changes in synaptic efficacy.

To confirm that astrocyte Ca^2+^ signal is necessary for the induction of c-LTP, we monitored SC synaptic transmission at single synapses using the minimal stimulation technique that activates single or very few presynaptic axons [11], and selectively elevated Ca^2+^ in astrocytes while preventing G protein-mediated signaling cascades in the astrocyte network (Figure 6A). For this purpose, astrocytes were whole-cell recorded and loaded with both 20 mM GDPβS, which prevented cholinergic-induced Ca^2+^ signal in astrocytes (Figure 3C–E), and the Ca^2+^-cage NP-EGTA (5 mM), which selectively and reliably elevates astrocyte Ca^2+^ after UV stimulation (Figure 6B) [11]. In agreement with previous results [7],[11], astrocyte UV Ca^2+^ uncaging evoked a transient potentiation of the probability of release (Pr) (*n* = 4 from 9 synapses; Figure 6D) that was abolished by MCPG (*n* = 4; not shown), which agrees with a presynaptic mGluR activation by Ca^2+^-dependent glutamate release form astrocytes. However, in these conditions, either Ca^2+^ uncaging or alveus TBS alone did not evoke long-term changes of SC synaptic transmission properties (measured 20 min after the stimuli; *n* = 9) (Figure 6C, 6E, and 6F). In contrast, pairing UV Ca^2+^ uncaging in astrocytes and alveus TBS induced LTP of Pr and the synaptic efficacy, without significantly affecting the synaptic potency (*n* = 9; see Materials and Methods) (Figure 6E and 6F), which is consistent with a presynaptic mechanism. Furthermore, LTP induced by simultaneous astrocyte Ca^2+^ uncaging and alveus TBS was prevented by MCPG (*n* = 6; Figure 6E and 6F), indicating that astrocyte Ca^2+^ elevations stimulate the release of glutamate that activates presynaptic mGluRs. Similar results were obtained when the different stimulus were delivered independently in different cells (Figure S2).

Taken together, these results indicate that cholinergic-induced LTP results from the temporal coincidence of postsynaptic activity and astrocyte Ca^2+^ elevations, which stimulate Ca^2+^-dependent glutamate release that activating mGluRs potentiate synaptic transmitter release (Figure 6G).

## Discussion

Present results obtained in vivo and in vitro show that hippocampal LTP evoked by cholinergic activity was associated with Ca^2+^ elevations in astrocytes, and that both phenomena were mediated by mAChRs. Unexpectedly, the cholinergic-induced LTP also required mGluR activation, which prompted us to evaluate the involvement of astrocytes because glutamate is a Ca^2+^-dependent released gliotransmitter [2]–[4],[7],[11],[23],[29]–[31]. Analysis of the underlying cellular mechanisms in hippocampal slices indicate that astrocyte Ca^2+^ elevations were necessary for the generation of this LTP, which could be elicited by pairing postsynaptic depolarizations and astrocyte Ca^2+^ uncaging (Figure 6; cf., [11]). Taken together, the present results indicate that cholinergic-induced LTP results from the temporal coincidence of postsynaptic and astrocyte Ca^2+^ activities simultaneously triggered by cholinergic axons, which stimulating Ca^2+^-dependent gliotransmission persistently potentiate synaptic transmitter release through activation of mGluRs. Present data provide the first demonstration in vivo that astrocytes are responsible for a specific physiological phenomenon of synaptic plasticity triggered by sensory stimuli, in which the astrocyte calcium signal and the release of the gliotransmitter glutamate are key elements.

Our results indicate that cholinergic activation of astrocytes stimulates the release of glutamate, which leads activating mGluRs to the long-lasting potentiation of synaptic transmission when coincident with a postsynaptic depolarization. Consistent with our previous work (see [7],[11]), we propose that these receptors are located presynaptically because this c-LTP results from the enhancement of transmitter release probability without changes in synaptic potency, which indicates a presynaptic rather than a postsynaptic underlying mechanism, and it is associated with changes in the paired-pulse facilitation (Figure S1), which is consistent with a presynaptic mechanism. Therefore, although anatomical evidence for presynaptic mGluRs at Schaffer collaterals needs to be confirmed, electrophysiological data strongly suggest that c-LTP is mediated by activation of presynaptic mGluRs.

The cholinergic-induced LTP requires the temporal coincidence of the astrocyte signaling and a mild postsynaptic depolarization, which suggests the existence of a retrograde signaling from the postsynaptic neuron to induce the presynaptic expression of LTP. Although further detailed studies are required to identify the possible postsynaptic signal and to elucidate their molecular targets, perhaps the presynaptic molecular events responsible for the astrocyte-induced mGluR-mediated transient potentiation of transmitter release (see Figure 6G; cf. [7],[11]) could become persistently altered by the signaling pathway stimulated by the postsynaptic signal.

The consequences of AChR activation on synaptic transmission and plasticity have been extensively studied [17]–[21],[25]–[28], and the ability of cholinergic signaling to induce LTP in hippocampal CA3-CA1 synapses is well known [20],[21],[26],[27]. Yet the requirement of mGluR activation was previously untested, probably because its involvement was logically unexpected. However, this participation is not surprising when considering the ability of astrocytes to respond to synaptic transmitters and to release gliotransmitters such as glutamate that can regulate synaptic transmission and plasticity, according to the Tripartite Synapse concept [2]–[4]. Thus, a well-known process newly examined considering the possible involvement of astrocytes is revealed to be mediated by novel unexpected mechanisms. Other physiological processes, whose underlying mechanisms are currently interpreted as overlooking the possible participation of astrocytes, might also provide novel unexpected results if revisited, as recently shown for hippocampal heterosynaptic depression [14] or endocannabinoid-mediated synaptic potentiation [7]. Nevertheless, possible novel mechanisms mediated by astrocytes might be rather additional than exclusive. Indeed, our results propose a novel mechanism underlying the cholinergic-induced LTP that may co-exist with the classical NMDAR-mediated LTP, which was pharmacologically blocked in our experimental conditions to isolate the studied phenomenon.

Compelling evidence provided by many laboratories has shown the relevance of the astrocyte Ca^2+^ signal and the gliotransmission in neurophysiology [2]–[8],[11],[14]. However, recent reports [33]–[35] have questioned their physiological importance based on negative results that failed to detect changes in synaptic transmission and plasticity using particular tests in transgenic animals, such as IP_3_R2^−/−^ mice. In contrast, here we provide evidence showing that both cholinergic-induced astrocyte Ca^2+^ signal and LTP are impaired in this type of transgenic mice. Therefore, astrocyte-synapse interactions are based on complex signaling processes that are not unselectively triggered by any type of stimulus and do not result in unspecific neuromodulation of any type of process; rather they probably depend on specific types of activity of particular circuits and synapses and cause neuromodulation of precise phenomena. Hence, these results further support the concept of the Tripartite Synapse that highlights the relevance of the astrocyte Ca^2+^ signal and the gliotransmission, and proposes a key role of astrocytes in synaptic physiology.

Astrocytes may regulate synaptic function through the release of different gliotransmitters, such as glutamate, ATP, or D-serine, with different neuromodulatory mechanisms and physiological consequences [3],[36]. The present study was designed to investigate the involvement of astrocytes in a particular phenomenon, the cholinergic-induced LTP. To isolate this particular phenomenon, in vitro experiments were conducted in the presence of NMDA and GABAergic receptor antagonists to prevent interferences from other astrocyte-neuron signaling and other possible synaptic plasticity mechanisms, such as the classical neuronal NMDAR-mediated LTP, the postsynaptic NMDA receptor activation by astrocytic glutamate [23], the D-serine-mediated NMDAR modulation involved in LTP [6], or the presynaptic NMDAR-mediated synaptic efficacy increase induced by astrocytic glutamate [13]. Although the present results indicate that NMDAR-mediated mechanisms are not required for the cholinergic-induced hippocampal LTP, additional mechanisms responsible for the involvement of astrocytes in synaptic plasticity may be present. These different mechanisms may be complementary rather than alternative, suggesting that different neuronal and astrocytic signaling processes may coexist, which would result in multiple mechanisms of synaptic plasticity that may be triggered under different network activities, thus providing a higher richness to the synaptic communication (see [36]).

The cholinergic system is involved in multiple brain functions, including learning and memory, as well as behavioral states [37]–[40]. The present results show a key role of astrocytes in cholinergic signaling, suggesting that astrocytes may directly participate in those brain functions. Furthermore, because dysfunctions of cholinergic transmission contribute to memory loss in some brain disorders such as Alzheimer's disease [41], present data suggest that astrocytes may be directly involved in these pathological states of the nervous system. Present data extend the classical Hebbian model for LTP based on the coincident pre- and postsynaptic activity, by including astrocytes as key cellular elements involved in the intercellular signaling occurring during synaptic function, and where the coincidence of astrocyte and postsynaptic activities evoked by a physiological process (i.e., cholinergic activity) induces long-term changes in synaptic efficacy.

In conclusion, the present findings show that the astrocyte Ca^2+^ signal is required for the generation of LTP in hippocampal synapses induced by cholinergic activity, indicating that astrocytes are necessary elements in some forms of synaptic plasticity and, hence, they are directly involved in memory processes and brain information storage.

## Materials and Methods

All the procedures for handling and sacrificing animals followed the European Commission guidelines (86/609/CEE).

### In Vivo Preparation

Adult Wistar rats (3 and 4 mo old; weigh: 180–300 g) and C57BL/6 and IP_3_R2^−/−^ mice (weigh: 45–60 g) were anesthetized with urethane (1.5 g/Kg and 1.8 g/Kg, respectively) and placed in a stereotaxic device. The body temperature was maintained at 37°C, and the end-tidal CO_2_ concentration was monitored.

### Hippocampal Slice Preparation

Hippocampal slices were obtained from Wistar rats (12–17 d old). In some cases, slices from C57BL/6 wildtype mice and IP_3_R2^−/−^ mice (13–18 d old), generously donated by Dr. J Chen, were used [32]. Animals were anaesthetized and decapitated. The brain was rapidly removed and placed in ice-cold artificial cerebrospinal fluid (ACSF). Slices (350–400 µm thick) were incubated during >1 h at room temperature (21–24°C) in ACSF that contained (in mM): NaCl 124, KCl 2.69, KH_2_PO_4_ 1.25, MgSO_4_ 2, NaHCO_3_ 26, CaCl_2_ 2, and glucose 10, and was gassed with 95% O_2_ / 5% CO_2_ (pH = 7.3). Slices were then transferred to an immersion recording chamber and superfused with gassed ACSF including 0.05 mM Picrotoxin and 5 µM CGP 55845 to block GABA receptors. To prevent possible NMDAR-mediated plasticity, experiments were performed in the presence of 50 µM AP5. Cells were visualized under an Olympus BX50WI microscope (Olympus Optical, Tokyo, Japan).

### Electrophysiology In Vivo

For rats, electrodes were placed stereotaxically according to [42]. Field potentials were recorded through tungsten macroelectrodes (1 MΩ) placed in the CA1 layer (A, −3.8; L, 1; V, 2.5 mm from Bregma). For mice, electrodes were placed stereotaxically according to [43]. Recording electrodes were placed at the CA1 area (1.2 mm lateral and 2.2 mm posterior to Bregma; depth from brain surface, 1.0–1.5 mm) and bipolar stainless steel stimulating electrodes aimed at the right Schaffer collateral–commissural pathway of the dorsal hippocampus (2 mm lateral and 1.5 mm posterior to Bregma; depth from brain surface, 1.0–1.5 mm). Extracellular excitatory postsynaptic field potentials (fEPSPs) were amplified (DAM80; World Precision Instruments, Sarasota, FL), bandpass filtered between 0.1 Hz and 1.0 kHz, and digitized at 3.0 kHz (CED 1401 with Spike 2 software; Cambridge Electronic Design, Cambridge, UK). SC fibers continuously stimulated with single pulses (100 µA, 0.3 ms, 0.5 Hz) using a bipolar stainless steel stimulating electrode (0.1 mm diameter) placed in the stratum radiatum (A, −3.8; L, 4; V, 4 mm from Bregma). The medial septum (A, −0.2; L, 0; V, 7 mm from Bregma) was stimulated using a similar electrode. The initial phase of the fEPSP was used to quantify SC synaptic transmission. To mimic theta activity (theta burst stimulation, TBS), the medial septum was stimulated with four trains at 5 Hz of 5 stimuli (at 40 Hz) delivered 10 times at 0.1 Hz.

### Electrophysiology in Slices

Electrophysiological recordings from CA1 pyramidal neurons and astrocytes located in the *stratum radiatum* were made using the whole-cell patch-clamp technique. Patch electrodes had resistances of 3–10 MΩ when filled with the internal solution that contained (in mM) for pyramidal neurons: KGluconate 135, KCl 10, HEPES 10, MgCl_2_ 1, ATP-Na_2_ 2 (pH = 7.3); and astrocytes were patched with 4–9 MΩ electrodes filled with an intracellular solution containing (in mM): MgCl_2_ 1, NaCl 8, ATP-Na_2_ 2, GTP 0.4 , HEPES 10, and either 40 mM BAPTA or 20 mM GDPβS, titrated with KOH to pH 7.2–7.3 and adjusted to 275–285 mOsm. Recordings were obtained with PC-ONE amplifiers (Dagan Corporation, Minneapolis, MN). Fast and slow whole-cell capacitances were neutralized and series resistance was compensated (≈70%). Recordings were rejected when the access resistance increased >20% during the experiment. Recordings from pyramidal neurons were performed in voltage-clamp conditions and the membrane potential was held at −70 mV to record SC-evoked EPSCs. During alveus TBS, recordings were performed in current-clamp conditions, unless stated otherwise (e.g., Figure 5C and 5D). Signals were fed to a Pentium-based PC through a DigiData 1440 interface board (Axon Instruments). The pCLAMP 10 software (Axon Instruments) was used for stimulus generation, data display, acquisition, and storage. Experiments were performed at room temperature (21–24°C).

To stimulate cholinergic axons, theta capillaries (10–30 µm tip; WPI, Sarasota, FL) filled with ACSF were used for bipolar stimulation. The electrodes were connected to a stimulator S-900 through an isolation unit (S-910, Dagan Corporation) and placed in the stratum oriens/alveus near the subiculum area (for simplicity herein termed alveus), which contains cholinergic axons from the diagonal band of Broca and septum [22],[44]. For TBS, four trains at 5 Hz of 5 stimuli (at 40 Hz) were delivered 10 times at 0.1 Hz. To stimulate SC fibers, electrodes were placed in the *stratum radiatum* of the CA1 region. Single pulses (250 µs duration) or paired pulses (50 ms interval) were delivered at 0.33 Hz. Basal EPSC values were recorded 10 min before the stimulus, and the relative mean amplitudes of 10 consecutive EPSCs from basal values were plotted over time (e.g., Figure 2F). Long-term changes of synaptic transmission were assessed from the relative amplitude of 30 consecutive EPSCs recorded 54–60 min after the stimulus (e.g., Figure 2G). Paired-pulse facilitation was quantified as PPF = [(2nd EPSC−1st EPSC)/1st EPSC].

For minimal stimulation of SC, the stimulus intensity (10–50 mA) was adjusted to meet the conditions that putatively stimulate a single, or very few, synapses (cf. [7],[11],[45],[46]) and was unchanged during the experiment. The recordings that did not meet these criteria [7],[11],[45],[46] and synapses that did not show amplitude stability of EPSCs were rejected. The synaptic current parameters analyzed were: synaptic efficacy (mean peak amplitude of all responses including failures), synaptic potency (mean peak amplitude of the successes), probability of release (Pr, ratio between number of successes versus total number of stimuli), and paired-pulse facilitation. The responses and failures were identified by visual inspection.

### Calcium Imaging In Vivo

Adult animals were craniotomized and the cortical tissue above the hippocampus was removed by aspiration to expose the dorsal hippocampus (see [47],[48]), which was bathed with 4 µl of Fluo-4 AM (2 mM) and sulforhodamine 101 (SR101, 125 µM), for 30–60 min and covered with 2% agar and a glass coverslip. Most of the Fluo-4-loaded cells were astrocytes as indicated by their SR101 staining (Figure 1A) (cf., [49],[50]). Cells were imaged with an Olympus FV300 laser-scanning confocal microscope. Ca^2+^ variations recorded at the soma of 5 to 11 astrocytes in the field of view were estimated as changes of the fluorescence signal over the baseline (ΔF/F0). Astrocytes were considered to respond to the stimulation when ΔF/F0 increased two times the standard deviation of the baseline during the stimulus or with a delay ≤15 s after the end of the stimulus, and the proportion of responding astrocytes in different conditions was compared.

### Calcium Imaging in Slices

Ca^2+^ levels in astrocytes located in the *stratum radiatum* of the CA1 region of the hippocampus were monitored by fluorescence microscopy using the Ca^2+^ indicator fluo-4 (Molecular Probes, Eugene, OR). Slices were incubated with fluo-4-AM (2–5 µl of 2 mM dye were dropped over the hippocampus, attaining a final concentration of 2–10 µM and 0.01% of pluronic) for 20–30 min at room temperature. In these conditions, most of the cells loaded were astrocytes [23], as confirmed in some cases by their electrophysiological properties [22],[51]–[53]. Astrocytes were imaged using a CCD camera (ORCA-235, Hamamatsu, Japan) attached to the microscope. Cells were illuminated during 100–500 ms with a xenon lamp at 490 nm using a monochromator Polychrome V (TILL Photonics, Gräfelfing, Germany), and images were acquired every 0.5–1 s. The monochromator and the camera were controlled and synchronized by the IP Lab software (BD Biosciences, MD) that was also used for quantitative epifluorescence measurements. Astrocyte Ca^2+^ levels were recorded from the astrocyte cell body and Ca^2+^ variations were estimated as changes in the fluorescence signal over the baseline. Astrocytes were considered to respond to the stimulation when Δ*F*/*F*0 increased two times the standard deviation of the baseline. In some cases, Ca^2+^ levels in single neurons or astrocytes were monitored by including 50 µM fluo-4 in the corresponding internal solution and recording pipette. The astrocyte Ca^2+^ signal was quantified from the probability of occurrence of a Ca^2+^ spike, which was calculated from the number of Ca^2+^ elevations grouped in 5-s bins recorded from 5 to 20 astrocytes in the field of view [7], and mean values were obtained by averaging different experiments. To test the effects of alveus stimulation on Ca^2+^ spike probability under different conditions, the respective mean basal (15 s before the stimulus start) and maximum Ca^2+^ spike probability (i.e., 5–10 s after) from different slices were averaged and compared. Local application of ACh (1 mM) was delivered by 30-s duration pressure pulses through a micropipette.

### Calcium Uncaging by UV-Flash Photolysis

In photo-stimulation experiments, single astrocytes were electrophysiologically recorded with patch pipettes filled with the internal solution containing (in mM): MgCl_2_ 1, NaCl 8, ATP-Na_2_ 2, GTP 0.4, HEPES 10, GDPβS 20, NP-EGTA 5, and 50 µM fluo-4 (to monitor Ca^2+^ levels). Ca^2+^ uncaging was achieved by delivering 10 trains at 0.1 Hz of 5 pulses (1-ms duration, 6–15 mW) at 5 Hz of UV light (340–380 nm) to the soma and processes of the recorded astrocyte (optical window of 15–25 µm diameter) using a flash photolysis system (Rapp OptoElectronic, Hamburg, Germany).

### Drugs and Chemicals

D-(-)-2-Amino-5-phosphonopentanoic acid (D-AP5), (S)-α-Methyl-4-carboxyphenylglycine (MCPG), (2S)-3-[[(1S)-1-(3,4-Dichlorophenyl)ethyl] amino-2-hydroxypropyl](phenylmethyl)phosphinic acid hydrochloride (CGP 55845), and 1,2-bis(2-aminophenoxy)ethane-*N*,*N*,*N*′,*N*′-tetraacetate (BAPTA) were purchased from Tocris Cookson (Bristol, UK). Fluo-4-AM, o-nitrophenyl EGTA, tetrapotassium salt (NP-EGTA), and sulforhodamine B were from Molecular Probes, Eugene, Oregon. All other drugs were from Sigma. For in vivo experiments, atropine sulfate (5 mg/kg) was intraperitoneally injected and its effects tested 10–15 min after the injection. For in vivo electrophysiological experiments, MCPG (100 nl, 1 mM) was injected into the hippocampus with a Hamilton microliter syringe. For in vivo Ca^2+^ imaging, MCPG (0.8 mM) was included in the solution bathing the dorsal hippocampus.

Data are expressed as mean ± s.e.m. Results were compared using a two-tailed Student's *t* test (α = 0.05). Statistical differences were established with *p*<0.05 (*), p<0.01 (**), and *p*<0.001(***).

## Supporting Information

Figure S1 Astrocyte-mediated c-LTP was associated with changes in paired-pulse facilitation index. (A) Representative mean EPSCs (10 consecutive traces) evoked by paired-pulse stimulation of SC before (basal) and 60 min after alveus TBS stimulation, and scaled trace of the basal EPSCs. (B) Summary of PPF index before (basal) and 60 min after alveus TBS (*n* = 10). *p<0.05. Data are presented as means ± s.e.m.
(TIF)

Figure S2 Astrocyte Ca^2+^ elevations induce LTP of transmitter release at single hippocampal synapses. (A–D) Relative changes in synaptic efficacy (i.e., mean amplitude of responses including successes and failures of neurotransmission), probability of neurotransmitter release (Pr), and synaptic potency (i.e., mean EPSC amplitude excluding failures) (bin width, 2 min) over time in basal non-stimulated slices (*n* = 5), UV-flash astrocyte stimulation (*n* = 6), alveus TBS (*n* = 5), and pairing both stimuli (*n* = 4). Zero time corresponds to the onset of the stimulation (UV Ca^2+^ uncaging and alveus TBS are depicted by arrows and horizontal bars, respectively).
(TIF)

## References

[1] Eroglu C, Barres B. A (2010). Regulation of synaptic connectivity by glia.. Nature.

[2] Halassa M. M, Haydon P. G (2010). Integrated brain circuits: astrocytic networks modulate neuronal activity and behavior.. Annu Rev Physiol.

[3] Perea G, Navarrete M, Araque A (2009). Tripartite synapses: astrocytes process and control synaptic information.. Trends Neurosci.

[4] Volterra A, Meldolesi J (2005). Astrocytes, from brain glue to communication elements: the revolution continues.. Nat Rev Neurosci.

[5] Di Castro M. A, Chuquet J, Liaudet N, Bhaukaurally K, Santello M, Bouvier D, Tiret P, Volterra A (2011). Local Ca(2+) detection and modulation of synaptic release by astrocytes.. Nat Neurosci.

[6] Henneberger C, Papouin T, Oliet S. H, Rusakov D. A (2010). Long-term potentiation depends on release of D-serine from astrocytes.. Nature.

[7] Navarrete M, Araque A (2010). Endocannabinoids potentiate synaptic transmission through stimulation of astrocytes.. Neuron.

[8] Panatier A, Theodosis D. T, Mothet J. P, Touquet B, Pollegioni L, Poulain D. A, Oliet S. H (2006). Glia-derived D-serine controls NMDA receptor activity and synaptic memory.. Cell.

[9] Panatier A, Vallée J, Haber M, Murai K. K, Lacaille J. C, Robitaille R (2011). Astrocytes are endogenous regulators of basal transmission at central synapses.. Cell.

[10] Pasti L, Volterra A, Pozzan T, Carmignoto G (1997). Intracellular calcium oscillations in astrocytes: a highly plastic, bidirectional form of communication between neurons and astrocytes in situ.. J Neurosci.

[11] Perea G, Araque A (2007). Astrocytes potentiate transmitter release at single hippocampal synapses.. Science.

[12] Porter J. T, McCarthy K. D (1996). Hippocampal astrocytes in situ respond to glutamate released from synaptic terminals.. J Neurosci.

[13] Santello M, Bezzi P, Volterra A (2011). TNFα controls glutamatergic gliotransmission in the hippocampal dentate gyrus.. Neuron.

[14] Serrano A, Haddjeri N, Lacaille J. C, Robitaille R (2006). GABAergic network activation of glial cells underlies hippocampal heterosynaptic depression.. J Neurosci.

[15] Fellin T, Halassa M. M, Terunuma M, Succol F, Takano H, Frank M, Moss S. J, Haydon P. G (2009). Endogenous nonneuronal modulators of synaptic transmission control cortical slow oscillations in vivo.. Proc Natl Acad Sci U S A.

[16] Halassa M. M, Florian C, Fellin T, Munoz J. R, Lee S. Y, Abel T, Haydon P. G, Frank M. G (2009). Astrocytic modulation of sleep homeostasis and cognitive consequences of sleep loss.. Neuron.

[17] Everitt B. J, Robbins T. W (1997). Central cholinergic systems and cognition.. Annu Rev Psychol.

[18] Madison D. V, Lancaster B, Nicoll R. A (1987). Voltage clamp analysis of cholinergic action in the hippocampus.. J Neurosci.

[19] Dudar J. D (1977). The role of the septal nuclei in the release of acetyl-choline from the rabbit cerebral cortex and dorsal hippocampus and the effect of atropine.. Brain Res.

[20] Auerbach J. M, Segal M (1994). A novel cholinergic induction of long-term potentiation in rat hippocampus.. J Neurophysiol.

[21] Fernández de Sevilla D, Buño W (2010). The muscarinic long-term enhancement of NMDA and AMPA receptor-mediated transmission at Schaffer collateral synapses develop through different intracellular mechanisms.. J Neurosci.

[22] Araque A, Martín E. D, Perea G, Arellano J. I, Buño W (2002). Synaptically-released acetylcholine evokes Ca^2+^ elevations in astrocytes in hippocampal slices.. J Neurosci.

[23] Perea G, Araque A (2005). Properties of synaptically evoked astrocyte calcium signal reveal synaptic information processing by astrocytes.. J Neurosci.

[24] Herreras O, Solís J. M, Muñoz M. D, Martín del Río R, Lerma J (1988). Sensory modulation of hippocampal transmission. I. Opposite effects on CA1 and dentate gyrus synapsis.. Brain Res.

[25] Hölscher C, Anwyl R, Rowan M. J (1997). Stimulation on the positive phase of hippocampal theta rhythm induces long-term potentiation that can be depotentiated by stimulation on the negative phase in area CA1 in vivo.. J Neurosci.

[26] Fernández de Sevilla D, Núñez A, Borde M, Malinow R, Buño W (2008). Cholinergic-mediated IP3-receptor activation induces long-lasting synaptic enhancement in CA1 pyramidal neurons.. J Neurosci.

[27] Leung L. S, Shen B, Rajakumar N, Ma J (2003). Cholinergic activity enhances hippocampal long-term potentiation in CA1 during walking in rats.. J Neurosci.

[28] Fernández de Sevilla D, Buño W (2003). Presynaptic inhibition of Schaffer collateral synapses by stimulation of hippocampal cholinergic afferent fibres.. Eur J Neurosci.

[29] Jourdain P (2007). Glutamate exocytosis from astrocytes controls synaptic strength.. Nat Neurosci.

[30] Navarrete M, Araque A (2008). Endocannabinoids mediate neuron-astrocyte communication.. Neuron.

[31] Shigetomi E, Bowser D. N, Sofroniew M. V, Khakh B. S (2008). Two forms of astrocyte calcium excitability have distinct effects on NMDA receptor-mediated slow inward currents in pyramidal neurons.. J Neurosci.

[32] Li X, Zima A. V, Sheikh F, Blatter L. A, Chen J (2005). Endothelin-1-induced arrhythmogenic Ca^2+^ signaling is abolished in atrial myocytes of inositol-1,4,5-trisphosphate(IP3)-receptor type 2-deficient mice.. Circ Res.

[33] Petravicz J, Fiacco T. A, McCarthy K. D (2008). Loss of IP3 receptor-dependent Ca^2+^ increases in hippocampal astrocytes does not affect baseline CA1 pyramidal neuron synaptic activity.. J Neurosci.

[34] Agulhon C, Fiacco T. A, McCarthy K. D (2010). Hippocampal short- and long-term plasticity are not modulated by astrocyte Ca^2+^ signaling.. Science.

[35] Agulhon C, Petravicz J, McMullen A. B, Sweger E. J, Minton S. K, Taves S. R, Casper K. B, Fiacco T. A, McCarthy K. D (2008). What is the role of astrocyte calcium in neurophysiology?. Neuron.

[36] Navarrete M, Araque A (2011). Basal synaptic transmission: astrocytes rule!. Cell.

[37] Blokland A (1996). Acetylcholine: a neurotransmitter for learning and memory?. Brain Res Rev.

[38] Deco G, Thiele A (2009). Attention: oscillations and neuropharmacology.. Eur J Neurosci.

[39] Hasselmo M. E (1999). Neuromodulation: acetylcholine and memory consolidation.. Trends Cogn Sci.

[40] Woolf N. J (1998). A structural basis for memory storage in mammals.. Prog Neurobiol.

[41] Kuchibhotla K. V, Lattarulo C. R, Hyman B. T, Bacskai B. J (2009). Synchronous hyperactivity and intercellular calcium waves in astrocytes in Alzheimer mice.. Science.

[42] Franklin G, Paxinos K (2001). The mouse brain in stereotaxic coordinates..

[43] Paxinos G, Watson C (1982). The Rat Brain in Stereotaxic Coordinates..

[44] Lewis P. R, Shute C. C. D (1967). The cholinergic limbic system: projections to hippocampal formation, medial cortex, nuclei of the ascending cholinergic reticular system, and the subfornical organ and supra-optic crest.. Brain.

[45] Dobrunz L. E, Stevens C. F (1997). Heterogeneity of release probability, facilitation, and depletion at central synapses.. Neuron.

[46] Isaac J. T. R, Hjelmstad G. O, Nicoll R. A, Malenka R. C (1996). Long-term potentiation at single fiber inputs to hippocampal CA1 pyramidal cells.. Proc Natl Acad Sci U S A.

[47] Kandel E. R, Spencer W. A, Brinley F. J (1961). Electrophysiology of hippocampal neurons. Sequential invasion and synaptic organization.. J Neurophysiol.

[48] Kuga N, Sasaki T, Takahara Y, Matsuki N, Ikegaya Y (2011). Large-scale calcium waves traveling through astrocytic networks in vivo.. J Neurosci.

[49] Hirase H, Qian L, Barthó P, Buzsáki G (2004). Calcium dynamics of cortical astrocytic networks in vivo.. PLoS Biol.

[50] Nimmerjahn A, Kirchhoff F, Kerr J. N, Helmchen F (2004). Sulforhodamine 101 as a specific marker of astroglia in the neocortex in vivo.. Nat Methods.

[51] Kang J, Jiang L, Goldman S. A, Nedergaard M (1998). Astrocyte-mediated potentiation of inhibitory synaptic transmission.. Nat Neurosci.

[52] Parri H. R, Gould T. M, Crunelli V (2001). Spontaneous astrocytic Ca^2+^ oscillations in situ drive NMDAR-mediated neuronal excitation.. Nat Neurosci.

[53] Nett W. J, Oloff S. H, McCarthy K. D (2002). Hippocampal astrocytes in situ exhibit calcium oscillations that occur independent of neuronal activity.. J Neurophysiol.

