# Supplementary figures and images for: Astrocytes Mediate In Vivo Cholinergic-Induced Synaptic Plasticity
Source: PLoS Biol. 2012 Feb 14;10(2):e1001259. doi: 10.1371/journal.pbio.1001259 (PMC3279365; doi:10.1371/journal.pbio.1001259)

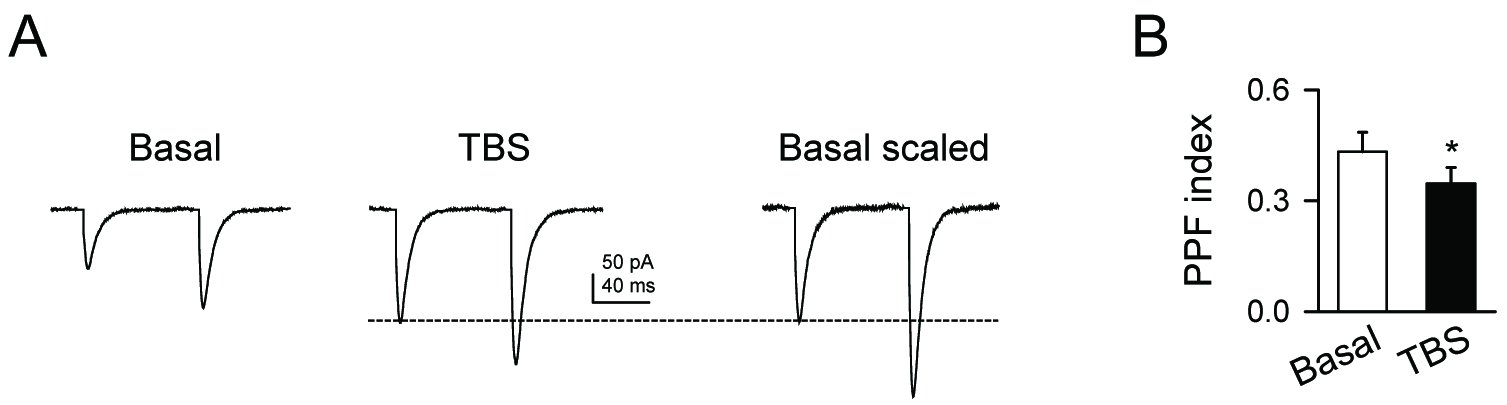

Supplement: Figure S1 — Astrocyte-mediated c-LTP was associated with changes in paired-pulse facilitation index. (A) Representative mean EPSCs (10 consecutive traces) evoked by paired-pulse stimulation of SC before (basal) and 60 min after alveus TBS stimulation, and scaled trace of the basal EPSCs. (B) Summary of PPF index before (basal) and 60 min after alveus TBS (n = 10). *p<0.05. Data are presented as means ± s.e.m. (TIF) [file pbio.1001259.s001.tif]

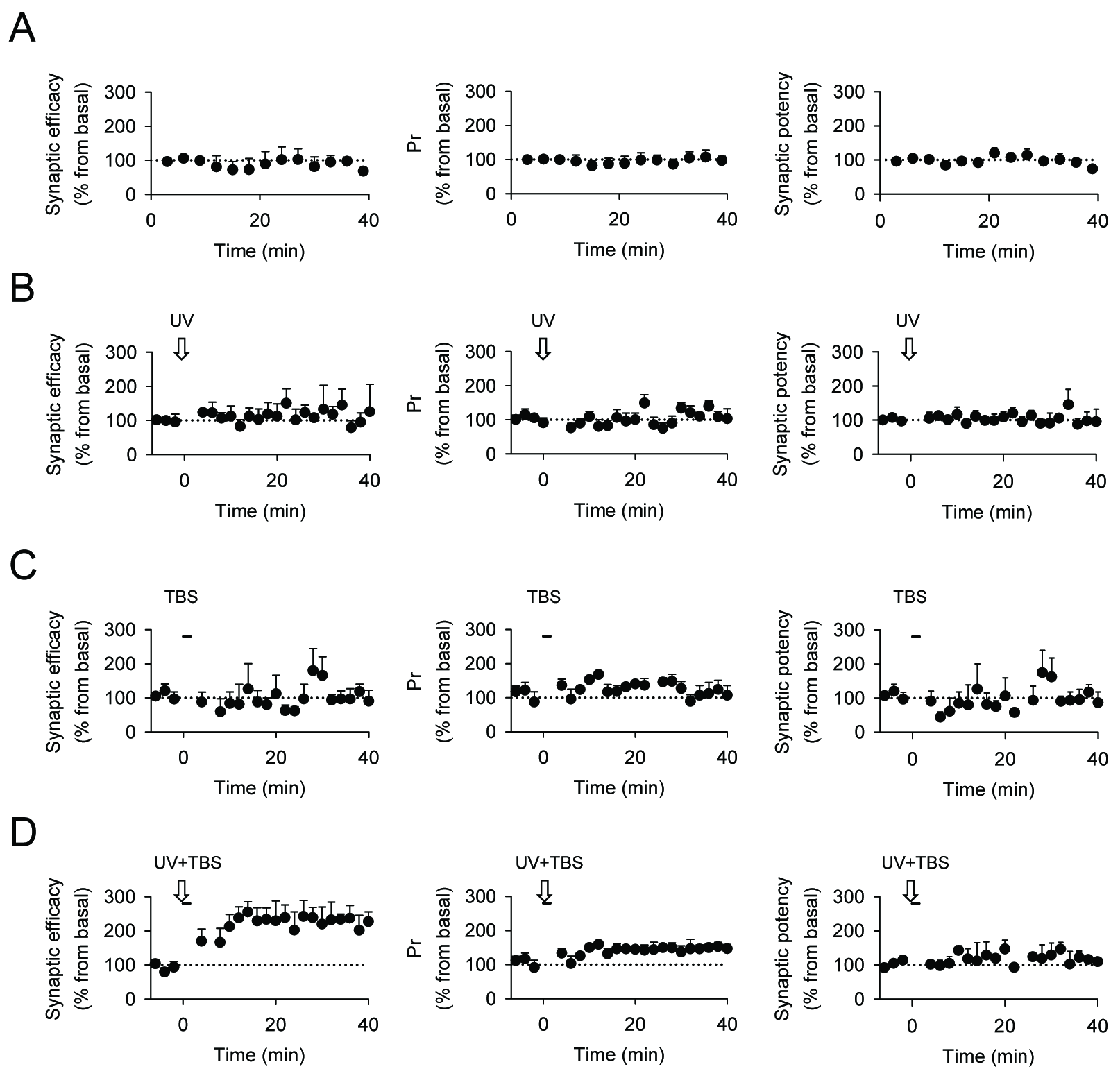

Supplement: Figure S2 — Astrocyte Ca2+ elevations induce LTP of transmitter release at single hippocampal synapses. (A–D) Relative changes in synaptic efficacy (i.e., mean amplitude of responses including successes and failures of neurotransmission), probability of neurotransmitter release (Pr), and synaptic potency (i.e., mean EPSC amplitude excluding failures) (bin width, 2 min) over time in basal non-stimulated slices (n = 5), UV-flash astrocyte stimulation (n = 6), alveus TBS (n = 5), and pairing both stimuli (n = 4). Zero time corresponds to the onset of the stimulation (UV Ca2+ uncaging and alveus TBS are depicted by arrows and horizontal bars, respectively). (TIF) [file pbio.1001259.s002.tif]
